# Medicinal Plants in Traditional Herbal Wines and Liquors in the East of Spain and the Balearic Islands

**DOI:** 10.3389/fphar.2021.713414

**Published:** 2021-09-29

**Authors:** V. Martínez-Francés, D. Rivera, C. Obon, F. Alcaraz, S. Ríos

**Affiliations:** ^1^ Biological Research Station-Botanical Garden of Torretes, Institute of Biodiversity CIBIO, University of Alicante, Alicante, Spain; ^2^ Departamento de Biología Vegetal, Facultad de Medicina, Universidad de Murcia, Murcia, Spain; ^3^ Centro de Investigación e Innovación Agroalimentaria y Agroambiental (CIAGRO), EPSO, Universidad Miguel Hernández de Elche, Alicante, Spain

**Keywords:** appetite stimulant, digestive, emmenagogue, ethnobotany, medicinal wines, medicinal liquor, tonic

## Abstract

Homemade herbal preparations from the East of Spain are the witness of traditional medicine inherited from the ancient complex formulas of herbal teas and medicinal wines. In this study, we document the use of traditional alcoholic beverages, identify their ingredients, almost exclusively botanical, record the local medicinal uses of these mixtures, and discuss patterns of distribution of this knowledge in regions of eastern Spain, the Balearic Islands and Andorra. We determine marker species and relevant patterns of herbal formulas in the different regions of the territory. Homemade liquors and liqueurs are consumed for their digestive and tonic-restorative properties but they also play in some cases an important social role. The elderly remember other medicinal uses such as aperitif, emmenagogue, or antidiarrheal, for some of the most popular preparations. The herbal liqueur formulas include predominantly Lamiaceae, Asteraceae, Rosaceae, Rutaceae, and Apiaceae species. Herbs (58%), fruits (28%), and mixtures of both (12%) are ingredients of liquors and wines, being the aerial parts the most frequent in terms of species (30%) and records (49%). *Dictamnus hispanicus, Santolina villosa, Salvia blancoana* subsp. *mariolensis*, *Rosmarinus officinalis, Thymus vulgaris,* and *Clinopodium serpyllifolium* subsp. *fruticosum* are the species most frequently used. Others species used to a lesser extent as *Polygonatum odoratum, Thymus moroderi,* and *Saxifraga longifolia* are restricted to locally homemade preparations because their collection and uses require special knowledge of the rare or endemic flora. Sustainability of these practices is strongly limited by the overall loss of local traditional knowledge and by the limited availability of most of the wild species; some of them are endangered or threatened mainly by the loss of their natural habitats. Cultivation and domestication are a promising alternative to collecting from wild populations. The cultivation of *Thymus moroderi* in the province of Alicante and *Polygonatum odoratum* in the province of Teruel are good examples. There is a notable decrease in the complexity of the formulas registered throughout the nearly 15 years of the study. This is interpreted as a consequence of a loss of knowledge, less accessibility to wild resources, and changes in traditions and preferences.

## Introduction

Hippocrates is credited for the sentence “let thy food be thy medicine and thy medicine be thy food.” This phrase is unlikely from Hippocrates; however, yet in line with the above philosophy, we are currently witnessing a reappraisal of the complementarity of nutrition and pharmacology (Witkamp and van Norren, 2018). The ethnobotany of wine has been studied recently, and ancient Egyptian and Phoenician wine residues reveal that these peoples added spices and healing herbs. Some healing plants whose remains were found in ancient Egyptian wines were coriander, lemon balm, peppermint, rosemary, sage, savory, senna, and thyme (Plotkin, 2021).

Soaking herbs for several days, weeks, or years in wine or distillates with different alcohol concentrations are a common practice in different cultures all over the world (Min and Jeong, 1995; Hwang et al., 2005; Wujisguleng et al., 2012; Egea et al., 2015; Egea et al., 2016). Their product is a wide range of extracts known as herbal wines and liquors (Egea et al., 2015).

The origin of liquors with medicinal plants, obtained by maceration in alcohols or by distilling wines, is difficult to determine but in any case it is the result of the development of distillation techniques (Forbes, 2009). The first trustworthy references for distilled alcohol date back to 1130 and 1160 CE at the Salerno School, where a certain Master Salernus distilled alcohol to make perfumes and medicines (Cierbide, 2007; Forbes, 2009). The first herbal liquors known as “*aqua ardens compositae*” appeared between the 13th and 14th centuries in the works of Salernitan scholars natives of the kingdom of Aragon such as Joanis de Rupescissa, Arnau de Villanova, and Raimond Llull (or their disciples) (Loring, 1993; Bueno and Alegre, 2001; Cierbide, 2007; Forbes, 2009; Cierbide, 2013; López-Pérez, 2016).

Spirits, often named liquor, contain no added sugar and at least 20% alcohol by volume, while liquors with added sugar and often herbs or flavorings are best known as liqueurs (Jurado, 2004; Egea et al., 2015).

The reasons for considering alcohol as medicinal are rooted in Galenism. According to the galenic principle “*contraria contrariis curantur*,” spirits which are hot and dry in third grade became a true panacea to cure all cold and wet diseases, especially gynecological disorders. Galenic “composite waters” combined the value of “hot” plants with alcohol (*aquae ardens* or *quinta esencia*, Rupecissa, 1346) as a remedy *per se* and solvent, vehicle, and preservative (García-Ballester, 2001; Cierbide, 2007). This *aqua vitae* was perceived closest to the *Elixir of life* so sought by alchemy (Martín-Reyes, 2004; López-Pérez, 2016). These liqueurs were called spirits, derived from the Greek concept of *pneuma* applied to medicine by Galen and assimilated to the product obtained from wine distillation as its spirit (Cierbide, 2007).

*Antidotarium Arnaldi* and *De Vinis,* both of Arnau de Villanova (McVaugh, 1993; Simó-Santonja, 2007; Gil-Sotres, 2011), recommended the use of herbal macerates, spiced wines, and spirits for gynecological disorders.

Traditional medical and alchemical knowledge in Spain is influenced by the Andalusian Muslim medicine (García-Ballester, 1984, 2001). This process was greater in the Kingdom of Valencia (García-Ballester, 1989; García-Ballester and McVaugh, 1996) through popularization of knowledge (García-Ballester, 1982; García-Ballester, 1984; García-Ballester, 2001) before the final expulsion of the Moors from Spain.

The greatest development of distillation and new medicines occurs in Spain from the second half of the 16th century, due to the support of King Felipe II and his court (Loring, 1993; Puerto, 1993; Bueno and Alegre, 2001; Martín-Reyes, 2004; López-Pérez, 2016). The greatest advances were made on this subject through the so-called “royal distillers” who worked in three great alchemy laboratories created and protected by the Crown in Aranjuez, Madrid, and El Escorial. One of them, Diego de Santiago, wrote a distillation manual *Arte separatoria*, and many distillation devices that were exported to other European courts (Bueno and Alegre, 2001). In 1592, Francisco Vallés following the orders of Felipe II tried to regulate the distillation of spirits throughout Spain, putting it exclusively in the hands of the apothecaries (Bueno and Alegre, 2001; Martín-Reyes, 2004). This law, which was a pioneer in Europe, implicitly recognizes that there were outside the court and its area of influence numerous particular industries that manufactured spirits to satisfy the great demand for distilled waters that existed among the population (Bueno and Alegre, 2001; Martín-Reyes, 2004).

Complex classical medical wines evolved into complex spirits or, as they are more popularly known, medicinal liquors. This is evidenced in the Castilian translations of Dioscorides by Laguna (1555), Laguna (1566) and in the first Catalan edition of 1617 from the *Libro de los Secretos de Agricultura, Casa de Campo y Pastoril* of Fray Miquel Agustín (Agustín, 1722).

This conceptual change will already remain invariant until the beginning of the 20th century. Today homemade and informal preparations produced locally at family level or small industries are known as traditional alcoholic beverages (Egea et al., 2016). The World Health Organization includes these traditional drinks in the so-called “unrecorded alcohol,” highlighting its cultural, social, and economic importance around the world, and the risk of their consumption. It has been estimated that almost one-quarter of all the alcohol consumed worldwide is drunk in the form of unrecorded alcohol (Rehm et al., 2014; WHO, 2018; Okaru et al., 2019). For many decades, home distillations have been disappearing. Currently, homemade liquor makers buy the liquor from local distilleries that already sell the legalized solvent to them.

In the East of Spain, most of these homemade liquors are elaborated, macerating some wild or cultivated plants in anise-flavored alcohol (Martínez-Francés and Ríos, 2005; Martínez-Francés et al., 2012; Martínez-Francés et al., 2015; Martínez-Francés et al., 2017). They are part of the traditional medicinal recipe repertory, inherited from the ancient complex formulas of herbal teas and medicinal wines (Martínez-Francés et al., 2015). The know-how linked to the elaboration of herbal liquors is part of the Traditional Knowledge System. This knowledge evolves usually over long periods of time and it is transmitted from one generation to the next (Hunn, 1998). The importance of this type of knowledge, defined as Intangible Cultural Heritage, was recognized by UNESCO in 2003 (Savo et al., 2011). The essence of Traditional Knowledge is not the mere product or use of a plant but includes a great sum of knowledge about the local environment and its ecological rules embracing culture, history, and symbolism. Nowadays, it is disappearing in industrialized countries as a consequence of different economic trends, food, and drug availability and changes in communication, culture, and values (Savo et al., 2011).

There are various general ethnobotanical studies in the different regions covered in this work with some partial references to traditional homemade herbal liquors: Aragon (Villar et al., 1992), Balearic Islands (Carrió and Vallès, 2012; Moll, 2003, 2005), Castilla-La Mancha (Fajardo et al., 2007; Verde et al., 2008), Catalonia (Agelet and Vallès, 2001; Bonet and Vallès, 2002; Parada et al., 2011; Figueras and Castelló, 2012), Murcia (Rivera et al., 2008), and Valencia (Mulet, 1991; Pellicer, 2001). But there are no works that cover the whole of the territory and are specifically dedicated to this matter that is on the interface on food and medicine.

The specific aims of the present research are as follows.

To document the formulation and uses, particularly those medicinal, of traditional homemade alcoholic beverages and the related knowledge in eastern Spain. For this, we intend to identify the different ingredients and their relative importance in the whole as well as determining the types and styles of these drinks and their geographic patterns if they exist.

It is also intended to analyze the evolution in the complexity of the registered formulas over the almost 15 years of study, discuss the factors that determine the patterns of geographic distribution of this knowledge, and, finally compare our results with available evidence from western Mediterranean areas on similar formulations reputed as nutraceuticals.

## Materials and Methods

### Study Area

**FIGURE 1:**
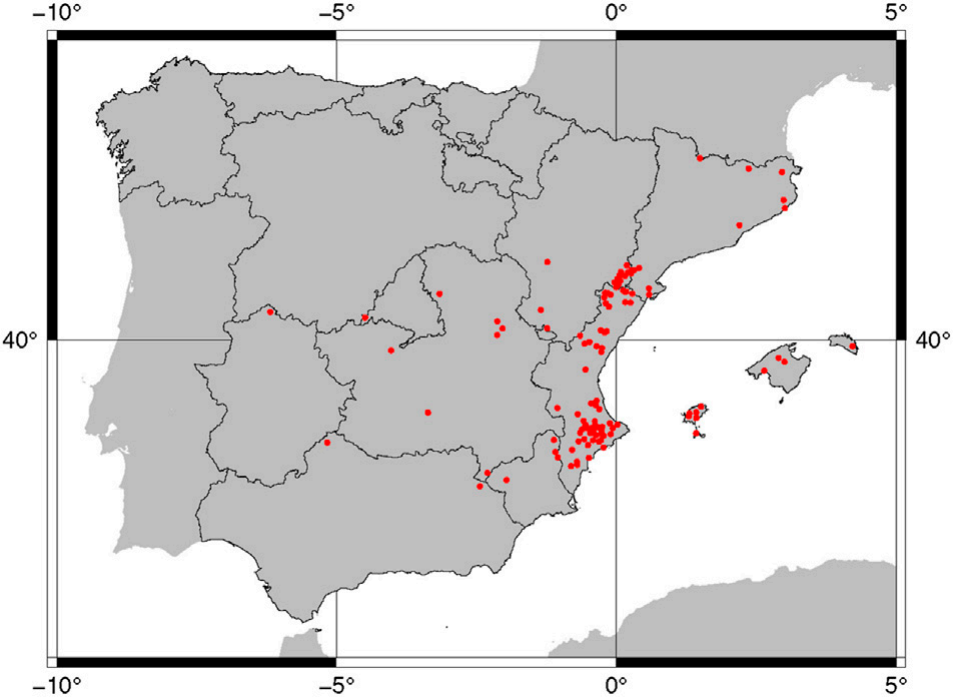
Location map of the studied areas.

The study area extends along eastern Spain including the Balearic Islands (Mallorca, Menorca, Ibiza, and Formentera Islands) and Andorra (Figure 1). The main sampling activities were developed within the Comunidad Valenciana (provinces of Alicante, Castellón, and Valencia), followed by southern Aragon (Teruel province) and southern Catalonia (Tarragona province). Castilla-La Mancha, Murcia, Madrid, Extremadura, and Andalusia region were less intensively sampled. We paid special attention to obtaining ethnobotanical data in the Valencian region and the Aragon border (Table 1). The rest of the areas have also prospected, although to a lesser extent, to follow our study methodology and to be able to compare the data.

**TABLE 1:** Geographical provenance of the formulas and samples analyzed.

Autonomous communities and countries	Provinces	Samples
Andalucia	(total)	2
Andorra^a^	(total)	3
Aragón	Teruel	56
	(total)	56
Baleares	(total)	26
Castilla-La Mancha	(total)	20
Cataluña	Tarragona	37
	(total)	55
Com. Valenciana	Alicante	239
	Castellón	105
	Valencia	48
	(total)	392
Extremadura	(total)	1
Madrid	(total)	1
Reg. Murcia	(total)	13

a Andorra is an independent country of the Pyrenees.

The provinces of Alicante, Valencia, and Castellón offer a homogeneous physiognomy from the general ethnological point of view. Murcia is a transition zone between Andalusia and the Levant, but socially and economically, Murcia is a Levantine land with Castilian influences. In both territories, the Moorish cultural footprint has persisted, especially in the garden areas along the rivers subject to traditional irrigation (Caro-Baroja, 1981). This Moorish persistence can also be seen in the Lower Aragón territories and the Teruel mountain ranges, singularly where fertile orchards are cultivated along the rivers (Caro-Baroja, 1981). Traditional housing is based on the use of local resources and is oriented to cope with extreme temperatures and the impact of torrential rains in the form of floods. Especially in the Valencian territory, the festivals are characterized by their ostentation and luxury and offer an exceptional occasion for the shared consumption of different herbal liqueurs or “herberos.” The population structure of the Balearic Islands differs from that of the Levantine coasts of the peninsula. However, the Valencian and Catalan influence, and even Aragonese, from the linguistic, social, and economic points of view, has been intensely felt in more modern times (Caro-Baroja, 1981). The Catalan territories are defined mainly by the Catalan language, which philologically differs from those of Valencia and the Balearic Islands, with which it shares a common substratum. Within the sampled area, the eastern Catalan dialect predominates in the provinces of Gerona and Barcelona and the western Catalan dialect in the south of Tarragona and the neighboring territories of Aragon (Caro-Baroja, 1981).

The whole of the study area lies within the Mediterranean Region, characterized by summer drought, which in the mountains is eventually attenuated by occasional storms (Peinado-Lorca and Rivas-Martínez, 1987). Most of the sampled localities fall, in terms of biogeography and vegetation, within the Valencian-Provençale-Balearic province and, especially, within the different sectors of the Valencian subprovince (57%) (Rivas-Martinez et al., 2014). However, other territories sampled in some detail belong to the Balearic Islands biogeographical subprovince (10%), Oro Iberian subprovince (c. 12%), and the Alicantese-Murcian sector (c. 9%) of the Murcian Almeriese province. Bioclimatically, most are, in terms of temperatures, Mesomediterranean (with average annual temperature 13–17°C) or Thermomediterranean (17–19°C) and in terms of rainfall are semiarid (200–350 mm), dry (350–600 mm), or subhumid (600–1,000 mm) (Peinado-Lorca and Rivas-Martínez, 1987).

The calcicolous thyme scrub, rosemary and *Cistus* garrigue, and holm oaks or junipers woodlands are the main formations in the Valencian subprovince (Folch, 1981; Folch et al., 1984; Costa, 1999) and where our informants obtain most of the ingredients along with fields and orchard gardens.

The geological and climatological diversity of this territory has generated numerous habitats, multiplying the plant diversity that has conditioned the population’s way of life. Mountain areas acquired a great reputation in terms of medicinal plant richness becoming centers of herbs collection and also as the most important places for peregrination (De-Terán, 1949).

### Data Collection and Plant Specimens Identification

Ethnobotanical fieldwork for this study was conducted along two main periods: the first, more intensive between 2005 and 2011, and a second period with less intensity later until 2018. Prior informed consent was obtained verbally before each interview, following the ISE Code of Ethics (ISE, 2006). We used a combined sampling strategy starting by defining a sampling frame focusing on inns, distilleries, and traditional “herberos” fairs in the main area of study and, then, recurred to the snowball sampling strategy when it was possible (Lambert, 1990; Turner, 2003).

Local knowledge for medicinal plants used in liquors and wines was collected from 83 female (170 records) and 194 male informants (412 records), using semistructured, focalized interviews (details are available in Supplementary Table S1). From the point of view of the languages and dialects (Burgueño, 2002; García-Mouton, 2007; Vales, 2012) spoken by our informants and in which the recipes were collected, Valencian prevails, followed by Spanish, Catalan of southern Catalonia, and Balearic. The occupations of our informants were varied, ranging from innkeeper (56%) or distiller (14%) to official or pharmacist (6% each), shepherd (4%), craft seller, farm shop keeper, honey seller, or folk group member (2% each) and forest ranger, tobacconist, rural tourism manager, healer, or priest (1% each).

It is worth noting that thirteen records were obtained in interviews that involved the joint participation of men and women from the same family unit. The estimated age of the interviewees at the interview moment ranged from 23 to 93. Based on the estimated age, only one informant was under 25; approximately 10% were between 25 and 39; the maximum number of informants occurred in the age ranges 40–54 (31%) and 55–69 (39%). Approximately 19% of our informants were 70–84 and only four were within the 85–99 age range.

Information on the herbs used and the way these were combined in each particular case was obtained from three main sources: first, and more often, the informants supplied us with the recipe or formula including the herbal ingredients and accompanied us to the field in order to verify the botanical identity of these when this was possible and to collect voucher specimens; alternatively, they gave us the bottle containing the liquor and the herbs and finally, which for us was the optimum, but more difficult to achieve, we obtained both recipe and liquor sample.

**FIGURE 2:**
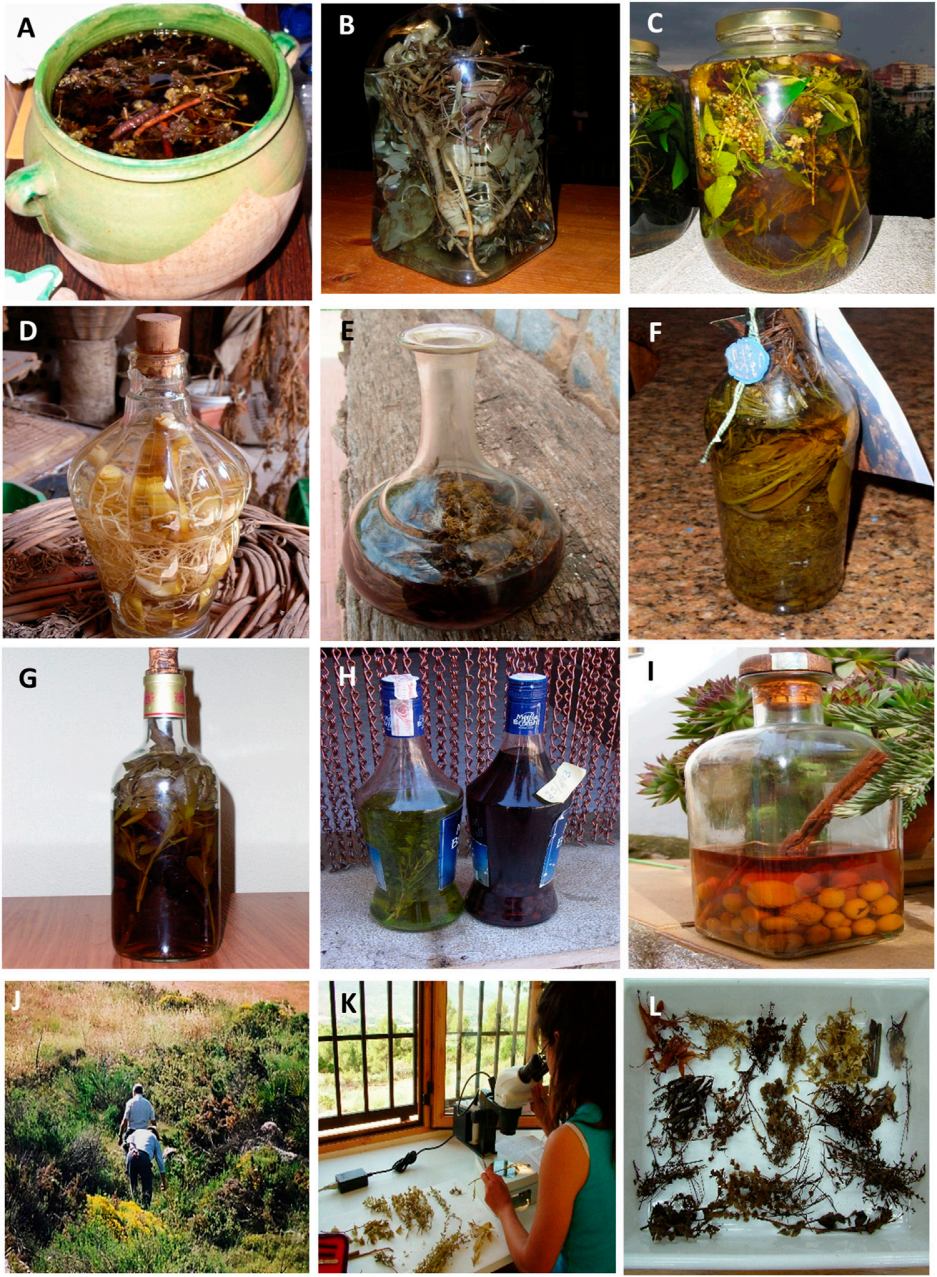
Main types of herbal liquors analyzed. **(A)** Herbero from Valencia region. **(B)** Beatamaria from Tarragona. **(C)** Ratafia from Girona. **(D)** Beatamaria from Teruel. **(E)** Cantueso from South of Alicante province. **(F)** Herbero from Formentera island. **(G)** Salvieta from North of Alicante province. **(H)** Gitam and fruit spirit from Castellon province. **(I)** Fruit spirit from Albacete province. **(J)** Collection of voucher specimens in the field accompanied with the informants. **(K,L)** Identification of bottle contents in the laboratory by a member of the research team.

Plant specimens were collected from field surveys, given, dried, by the informants, or obtained by the analyses of samples (bottles with medicinal wines or liquors) (Figure 2; Table 2). They were identified using the Flora dels Països Catalans (Bolòs and Vigo 1984; Bolòs and Vigo, 1989; Bolòs and Vigo, 1996; Bolòs and Vigo, 2001) and, in some cases, with the help of specialists in floristic and systematic botany. Voucher specimens were deposited in the Herbarium of the Barcelona University (BCN) and the Herbarium of the Valencia University (Jari Botanic) (VAL) (details are available in Supplementary Table S2). The material obtained alive in good condition for cultivation was introduced in the living collections of the Torretes Botanical Garden and used to prepare voucher specimens. Plant nomenclature is in accordance with POWO (POWO, 2021). For foreign or cultivated species determination, we followed Rivera et al. (1996); Rivera et al. (1998) and actualized their nomenclature according to GRIN (2021). The species were identified by Martinez-Frances and Rios.

**TABLE 2:** Main types of information recorded and their regional frequency.

Region	Only formulas	Only liquor samples	Formula and sample	Totals
Castilla-La Mancha	8	2	10	20
Com. Valenciana	268	54	78	400
Andorra^a^	3	0	0	3
Cataluña	28	1	18	47
Extremadura	0	0	1	1
Andalucia	2	0	0	2
Baleares	17	5	4	26
Madrid	1	0	0	1
Reg. Murcia	11	0	2	13
Aragón	49	2	5	56
Total	377	64	118	569

a Andorra is an independent country of the Pyrenees.

### Data Management and Analysis

The data collected during the fieldwork refer to the preparation and consumption of any type of homemade herbal alcoholic beverage. These data have been entered in a database generated with Microsoft Excel^®^ 2007. These include, among others, information on plant ingredients (scientific name, family, vernacular name/s, and plant parts used), type of solvent (distillate, anise-flavored alcohol, and wine), type of beverage (distillate, liquor, and aromatized wine), main herbal types (fruits, herbs, mixture of fruits and herbs, fungus, and animal), processing and main consumption contexts and patterns, current usage, and medicinal local uses (digestive, aperitif, tonic-restorative, antidiarrheal, emmenagogue, and preservative-wine). Each elemental record represents one citation, defined as a single reported use for a single plant by a single informant. Different records are considered those that differ from each other in at least one of the following data: species, plant parts used, informant, type of beverage, and use category. We used for the analysis of data the set of functions and formulas available in Microsoft Excel^®^ 2007. These include the creation of thesaurus to standardize scientific names of plants and names of regions and provinces using VLOOKUP. Among other functions frequently used for this study are SUMIF and COUNTIF.

Given the extraordinary diversity of the sample in terms of informants (277), localities (126), different formulas (533), and identified ingredients (215 species), the dissimilarities calculated through the Sokal-Sneath index (Perrier et al., 2003), based on the presence/absence using Darwin 6 (Perrier and Jacquemoud-Collet, 2006), resulted in non-Euclidean distances, with low resolution. In the factorial analysis, only 7% of inertia was explained by the first axis. Therefore, we considered that the use of multivariate analysis techniques would not be appropriate for the study of the group that is the object of this work.

Maps have been created with The Generic Mapping Tools^©^ 1991–2020, version 6.1.1 [64-bit] (GMT, 2021).

Main differences in the type of homemade beverages taken into account in this study consist in the ingredients macerated (species and part of the plant) and the type of solvent used (Table 3). For liquors, anise-flavored alcohol (Martínez-Francés and Ríos, 2005; Martínez-Francés et al., 2012; Martínez-Francés et al., 2017) is usual. The alcoholic concentration of spirits has been noted and classified considering two categories: one between 20 and 35° and the other between 36 and 70°. In many cases, this is the difference between spirits bought in warehouses, the former, or made in their own alembics, the latter. The origin of alcohol (*Vitis vinifera* L., *Beta vulgaris* L. subsp. *vulgaris* Sugar beet group, and *Saccharum officinarum* L.), the sweetener (*B. vulgaris* subsp. *vulgaris*, *S. officinarum*, and honey), and the anise flavoring (*Illicium verum* Hook.f. and *Pimpinella anisum* L.) has also been identified wherever possible. However, these were not included in our analysis since they were not part of the macerate mixtures and, therefore, did not yielded voucher specimens. In the case of aromatized wines, the most common base used is red wine, although in some cases, white or rose wines have also been used.

**TABLE 3:** Ethanol contents of the bases used for macerating the plant ingredients and sources for this alcohol.

**A**	
**Alcohol contents in percentage**	Formulas and liquor samples
Wine (16–20%)	63
Low alcohol spirit (20–34%)	312
Aniseed spirit and others (35–70%)	194
**B**	
**Source and type**	Formulas and samples
Wine/distilled wine alcohol	289
Honey	9
Distilled molasses alcohol	298
Spirit aromatized with “*anís de matalauva*” or star anise	278

Often the final liquor is elaborated by mixing different bases; hence, the total can exceed the number of 569 formulas studied.

Those species that serve as a base for the alcoholic beverage, as sweeteners, or as flavorings and are previous to the addition of the herbs to macerate are excluded from the total species count of each recipe.

## Results

### General Results

We interviewed 277 informants (194 men; 83 women) whose average age was 56 years. Men account for 70% of those interviewed, highlighting the importance of this aspect in the preparation of medicinal liquors, compared to the usual role of women in family care.

In the first interview period, until 2011, older people were prioritized, reaching 23% of those interviewed, with an age between 70 and 93 years. The most numerous age group studied is between 40 and 70 years, representing 66% of the total. In the second period, until 2018, the interviews with younger people were intensified. This was the period when some of them replaced, in the liquor preparation activities, the older informants of their families.

The approximate age of the informants is relevant for the number of formulas recorded, with a maximum of 227 records from informants between 40 and 54 years, followed by 166 from those between 55 and 69 and 113 from those aged 70 to 84.

The bases used for macerating the plant ingredients are wines (usually red wine) and spirits of different ethanol concentrations (Table 3).

We compiled an inventory of 215 taxa from 56 families. Most, 128 (60%), are wild species, followed by 74 cultivated (34%), few are imported (12, 6%), and one is feral. The proportion of imported species differs along the types and styles recorded. Vascular plant taxa are 212, two are fungi, and one is zoological.

From a phytogeographic perspective (POWO, 2021), Mediterranean species predominate (118, 55%) followed by those European (37, 17%) and widespread (30, 14%). Minor represented groups include Northern Hemisphere, Asia, America, Southern Hemisphere, and Pacific Islands.

### Medicinal Uses of the Herbal Wines and Spirits

Almost 87% of the recorded formulations are reputed by our informants as being digestive and are consumed as such (Table 4). However, they recognized that also most herbal liquors were consumed in social events involving high food intake or, especially by the younger population, merely “per se” no matter the supposed health benefits of their consumption. The use as a general tonic or to improve their mood along with appetite stimulant follows in importance (Table 4). Their uses as antidiarrheal (especially Balearic *herberos*) or as emmenagogue (notably *beatamaria*) are worthy of notice, although in terms of percentage they are low (below 10% each).

**TABLE 4:** Medicinal and other uses of the studied herbal wines and spirits.

Main use	Formulas and samples	%
Medicinal		
• Digestive	493	86.6
• Tonic (restorative)	178	31.3
• Aperitif	78	13.7
• Antidiarrheal	53	9.3
• Emmenagogue	49	8.6
Others		
• Social	440	77.3
• Preservative (wine)	26	4.6

Liquors are often consumed for more than one purpose (cf. Table 6); hence, the totals can exceed the number of 569 formulas studied.

The eldest informants are those who have described a greater diversity of medicinal uses for wines and liquors, also referring to the use of the same formulas in herbal teas on a regular basis. For them, some liquors are multipurpose, reporting even four or five different uses for one single liquor (twenty-nine and seven formulas, respectively). However, the commonest figure recorded is two or three different uses (340 and 147 formulas, respectively). Therefore, the largest number of references recorded present two uses: to be consumed in social events combined with a single medicinal purpose. Only forty-five formulas were reported to have a single one use (medicinal or not).

### The Ingredients

Aerial parts are the most frequently used plant parts (Table 5) followed by leaves, flowers, and fruits.

**TABLE 5:** Frequency of plant parts among the analyzed liquors.

Parts	Species	%	Records^a^	%
Aerial parts	70	30.0	1,279	49.3
Leaves	45	19.3	423	16.3
Flowers/inflorescences	35	15.0	250	9.6
Fruits	31	13.3	257	9.5
Tender shoots	22	9.4	53	2.0
Roots	7	3.0	46	1.8
Seeds	5	2.1	37	1.4
Stems	4	1.7	118	4.6

a Note that one single sample with different plant species can contain more than once the different recognized parts.

Lamiaceae is by far the most widely represented plant family in terms of species (c. 28%) but slightly surpassed by Rutaceae in the percentage of samples (Table 6). Asteraceae follows in the percentage of samples notwithstanding its high number of species in the Spanish flora (Mateo and Crespo, 2014; Anthos, 2021). We must underline the overrepresentation of Rutaceae in the formulas that are due in the case of cultivated *Citrus* species to their aromatic leaves, flowers, and fruits and the abundance of orange, lemon, and tangerine orchards in, notably, Valencia and Murcia regions. Wild Rutaceae species are noticeable because nevertheless their relative scarcity in the area, they are highly sought for with the purpose of elaborating liqueurs (Table 6).

**TABLE 6:** Plant families more often present in the analyzed liquors.

Plant family	Samples	%	Species	%
Rutaceae	244	42.9	11	5.1
Lamiaceae	240	42.2	60	27.9
Asteraceae	177	31.1	20	9.3
Apiaceae	154	27.1	11	5.1
Rosaceae	86	15.1	20	9.3
Verbenaceae	86	15.1	1	0.5
Rubiaceae	57	10.0	5	2.3
Lauraceae	53	9.3	2	0.9
Juglandaceae	44	7.7	1	0.5
Malvaceae	38	6.7	4	1.9
Myrtaceae	28	4.9	3	1.4
Vitaceae	26	4.6	1	0.5
Hypericaceae	24	4.2	3	1.4
Asparagaceae	22	3.9	1	0.5
Cistaceae	9	1.6	7	3.3

Among the 215 species identified, two are fungi, one is Animalia, and 212 are Plantae.

Of the 56 families of plants, animals or fungi identified, only the fifteen with more than five species or that appear in more than 20 samples are represented in this table and they are all plants.

Among the 215 species identified, only 21 were recorded as ingredients of more than 40 formulas (Table 7). Given the high number of *herberos* analyzed, their contribution to the list of relevant species is significant.

**TABLE 7:** Most frequently species in the liquors analyzed from E Spain.

Species	Frequency (samples)	%
*Dictamnus hispanicus* Webb ex Willk.	183	32.2
*Santolina villosa* Mill.	114	20.0
*Salvia blancoana* subsp. *mariolensis* Figuerola	103	18.1
*Rosmarinus officinalis* L.	100	17.6
*Thymus vulgaris* L. subsp. *vulgaris*	97	17.0
*Clinopodium serpyllifolium* subsp. *fruticosum* (L.) Bräuchler	90	15.8
*Aloysia citriodora* Palau	86	15.1
*Foeniculum vulgare* Mill. subsp. *piperitum* (Ucria) Cout.	81	14.2
*Sideritis hirsuta* L.	79	13.9
*Sideritis tragoriganum* Lag. subsp. *tragoriganum*	74	13.0
*Citrus* x *limon* (L.) Osbeck	67	11.8
*Eryngium campestre* L.	63	11.1
*Teucrium capitatum* subsp. *gracillimum* (Rouy) Valdés Berm.	59	10.4
*Melissa officinalis* L.	54	9.5
*Thymus moroderi* Pau ex Martínez	52	9.1
*Cinnamomum verum* J.Presl	46	8.1
*Stachys heraclea* All.	46	8.1
*Coffea arabica* L.	45	7.9
*Mentha pulegium* L.	45	7.9
*Juglans regia* L.	44	7.7
*Chiliadenus glutinosus* (L.) Fourr.	41	7.2

Among the 215 recorded species, those 21 species found in more than 40 samples are only represented.

Lamiaceae is not only the most relevant plant family in the number of species but also in the frequency they are used. Among the 21 most commonly used plant species, over one-half are Lamiaceae (Table 7). Among these, *Salvia blancoana* subsp. *mariolensis, Rosmarinus officinalis, Thymus vulgaris*, and *Clinopodium serpyllifolium* subsp. *fruticosum* are present in more than 15% of the formulas.

A few species furnish more than one ingredient which are separately used by our informants. Those supplying three or more ingredients are listed in Table 8. Three Rutaceae are included, *Citrus* x *limon*, *C.* x *sinensis*, and *Dictamnus hispanicus*. These three species, together with *Foeniculum vulgare* subsp. *piperitum*, stand out for their popular role as flavoring in foods.

**TABLE 8:** Species furnishing more than two ingredients.

Standardized species	Number of ingredients	Ingredients
*Citrus* x *limon* (L.) Osbeck	5	Tender shoots, flowers/inflorescences, fruit rind, leaves, and extract (juice)
*Foeniculum vulgare* Mill. subsp. *piperitum* (Ucria) Cout.	4	Aerial parts, leaves, stems, and seeds
*Citrus* x *sinensis* (L.) Osbeck	4	Flowers/inflorescences, fruits, fruit rind, and leaves
*Dictamnus hispanicus* Webb ex Willk.	3	Aerial parts, leaves, and roots
*Artemisia absinthium* L.	3	Aerial parts, tender shoots, and extract (essential oil)
*Juniperus oxycedrus* L.	3	Tender shoots, leaves, and cones
*Rubus ulmifolius* Schott	3	Tender shoots, fruits and flowers/inflorescences
*Pinus halepensis* Mill.	3	Tender shoots, leaves, and flowers/inflorescences

Among the 36 species furnishing more than one ingredient (depending on the plant part used), only those furnishing three or more ingredients are represented.

**FIGURE 3:**
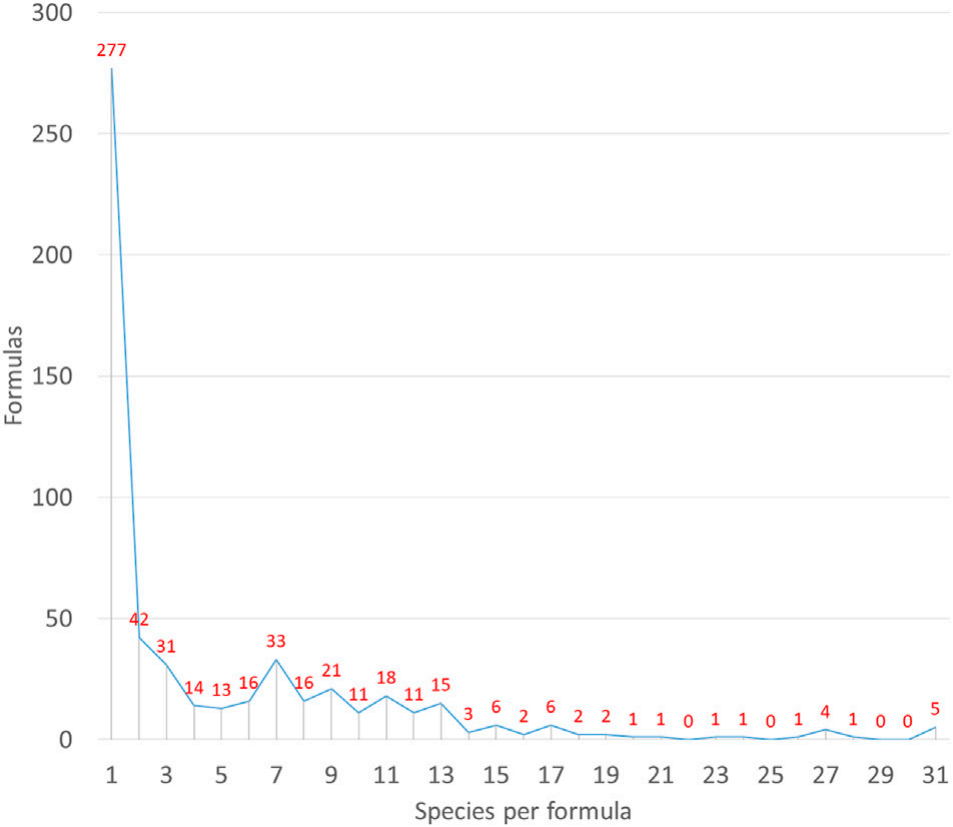
Complexity of formulations in terms of number of species entering the formulas.

Regarding the number of ingredients macerated in the base alcohol for the elaboration of the liquors, it varies from one to more than 30 (Figure 3). The maximum diversity records for this study are 48 species in a ratafia recipe from Girona province and 47 in one herbero from Mallorca Island.

### Main Types and Styles Based on the Mixture of Herbs

**FIGURE 4:**
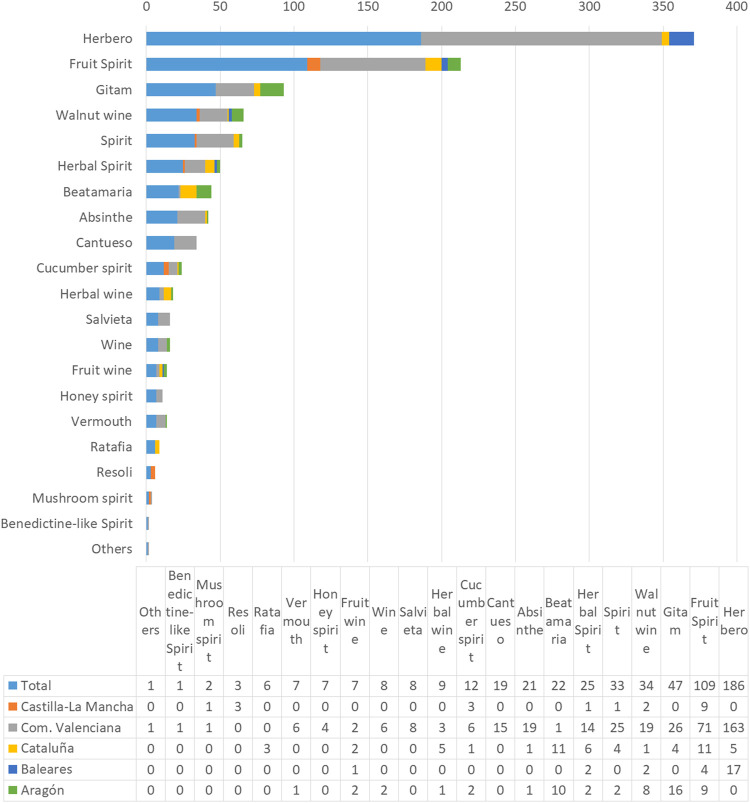
Main types of herbal liquors analyzed and their frequencies.

We have recorded 569 formulas and/or samples of wines, spirits, and liquors (Table 2). Among these, 423 are unique differing in their combination of ingredients. These were classified according to their solvent and herbal ingredient composition into the following main types: absinthe, *beatamaria*, *cantueso*, cucumber spirit, fruit spirit, fruit wine, *gitam*, herbal spirit, herbal wine, *herbero*, honey spirit, mushroom spirit, *ratafia, resoli, salvieta*, spirit, vermouth and walnut wine, and Benedictine-like and digestive wine. Their relative frequencies and distribution in the different regions studied show peculiar patterns that are summarized in Figure 4. The most relevant species and their combinations allow defining these peculiar types of herbal liquors (Table 9).

**TABLE 9:** Regional styles based on ingredient frequencies in the percentage of regional samples.

Standardized species	Baleares	Com. Valenciana	Aragón	Cataluña	Castilla-La Mancha	Reg. Murcia
*Dictamnus hispanicus* Webb ex Willk.	0.00	37.56	28.57	18.18	0.00	15.38
*Santolina villosa* Mill.	23.08	26.42	0.00	10.91	0.00	0.00
*Salvia blancoana* subsp. *mariolensis* Figuerola	0.00	24.35	0.00	0.00	0.00	0.00
*Thymus vulgaris* L. subsp. *vulgaris*	3.85	23.83	0.00	5.45	0.00	7.69
*Clinopodium serpyllifolium* subsp. *fruticosum* (L.) Bräuchler	0.00	21.50	0.00	7.27	5.00	0.00
*Sideritis hirsuta* L.	0.00	20.47	0.00	0.00	0.00	0.00
*Rosmarinus officinalis* L.	50.00	19.69	0.00	16.36	0.00	0.00
*Sideritis tragoriganum* Lag. subsp. *tragoriganum*	0.00	19.17	0.00	0.00	0.00	0.00
*Aloysia citriodora* Palau	53.85	15.80	0.00	14.55	0.00	0.00
*Teucrium capitatum* subsp. *gracillimum* (Rouy) Valdés Berm.	0.00	15.28	0.00	0.00	0.00	0.00
*Foeniculum vulgare* Mill. subsp. *piperitum* (Ucria) Cout.	7.69	15.03	0.00	0.00	0.00	0.00
*Thymus moroderi* Pau ex Martínez	0.00	12.69	0.00	0.00	0.00	23.08
*Juglans regia* L.	11.54	5.96	14.29	5.45	10.00	15.38
*Pimpinella anisum* L.	7.69	5.18	0.00	5.45	15.00	15.38
*Cinnamomum verum* J.Presl	11.54	6.99	3.57	12.73	20.00	15.38
*Vitis vinifera* L.^a^	0.00	3.63	3.57	3.64	20.00	7.69
*Syzygium aromaticum* (L.) Merr. and L.M.Perry	0.00	1.81	0.00	10.91	15.00	7.69
*Citrus* x *limon* (L.) Osbeck	46.15	9.33	1.79	7.27	0.00	0.00
*Santolina magonica* (O.Bolòs, Molin. and P.Monts.) Romo	38.46	0.00	0.00	0.00	0.00	0.00
*Foeniculum vulgare* Mill. subsp. *piperitum* (Ucria) Cout.	38.46	0.26	0.00	10.91	0.00	0.00
*Thymbra capitata* (L.) Cav.	34.62	0.52	0.00	7.27	0.00	0.00
*Citrus* x *sinensis* (L.) Osbeck	23.08	2.85	0.00	5.45	15.00	7.69
*Ruta chalepensis* L.	23.08	0.00	0.00	7.27	0.00	0.00
*Melissa officinalis* L.	15.38	11.40	0.00	5.45	0.00	0.00
*Coffea arabica* L.	15.38	8.29	0.00	1.82	20.00	7.69
*Juniperus oxycedrus* L.	19.23	0.00	0.00	7.27	0.00	0.00
*Mentha spicata* L.	19.23	5.18	3.57	3.64	0.00	0.00
*Salvia microphylla* Kunth	19.23	3.37	0.00	0.00	0.00	0.00
*Citrus* x *sinensis* (L.) Osbeck	19.23	0.78	0.00	3.64	0.00	0.00
*Citrus* x *limon* (L.) Osbeck	15.38	1.55	1.79	12.73	0.00	7.69
*Laurus nobilis* L.	15.38	0.52	0.00	3.64	0.00	0.00
*Pinus halepensis* Mill.	15.38	0.00	0.00	0.00	0.00	0.00
*Polygonatum odoratum* (Mill.) Druce	0.00	0.26	17.86	20.00	0.00	0.00

Ingredients with a frequency of 15% or higher within at least one regional ensemble are only represented here.

a Raisins.

The complexity of the formulations recorded is extremely variable between the different types and even within each type. *Herbero* with 47 and *ratafia* with 48 present the maximum number of ingredients within a single formulation. However, within these same types, we have found simpler formulations with five or fewer ingredients. The rest of the types have a number of ingredients oscillating between one and nine. *Absenta, beatamaria, cantueso, gitam,* and *salvieta* are noteworthy herbal liquors simple or with lesser ingredient diversity.

**FIGURE 5:**
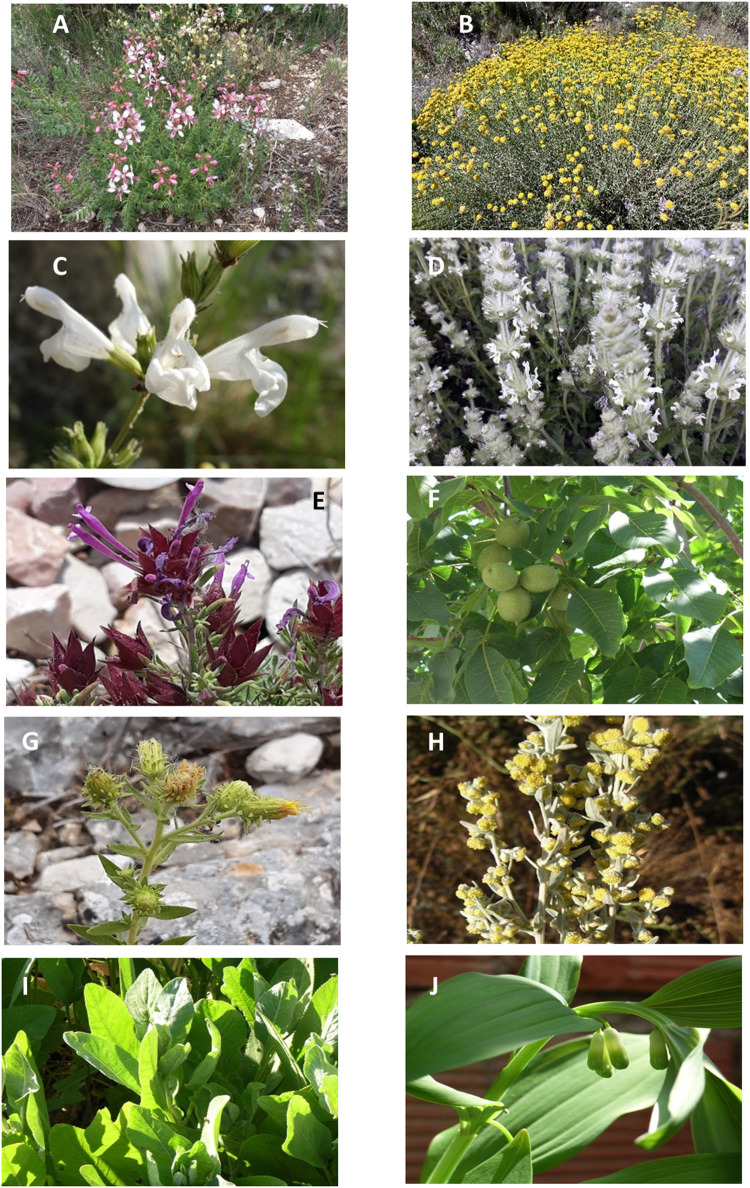
Plant species characteristic of the different types and styles of liquors. **(A)** *Dictamnus hispanicus* (herbero and gitam). **(B)** *Santolina villosa* (herbero). **(C)** *Salvia blancoana* subsp. *mariolensis* (salvieta). **(D)** *Sideritis hirsuta* (herbero). **(E)** *Thymus moroderi* (cantueso). **(F)** *Juglans regia* (Walnut wine). **(G)** *Chiliadenus glutinosus* (Herbal wines). **(H)** *Artemisia absinthium* (Absinthe and Vermouth). **(I)** *Tanacetum balsamita* (herbero). **(J)** *Polygonatum odoratum* (beatamaria).

*Polygonatum odoratum* characterizes the *beatamaria* formulations of Aragon and Catalonia (Table 9). It is possible to find simple and complex variants of the main formula, being always the core species *Polygonatum odoratum* (Figure 5)*. beatamaria* is elaborated in a small area where coincide three provinces, Tarragona (Catalonia), Teruel (Aragon), and Castellón (Valencia region).

**FIGURE 6:**
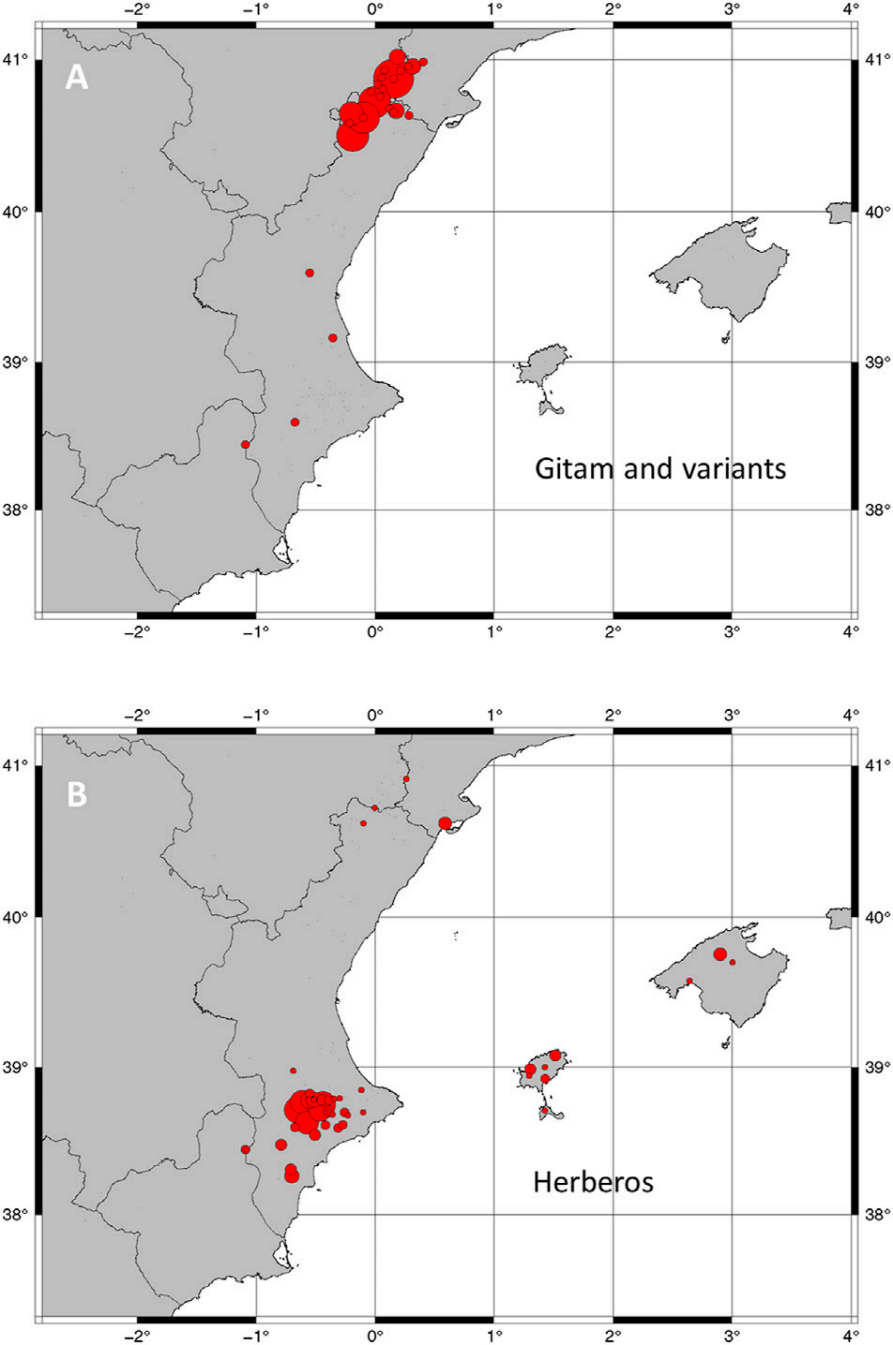
Geographical distribution of the two major types of liquors. **(A)** Gitam, *Dictamnus hispanicus* liquors. **(B)** Herberos. Note: dimensions of circles are proportional to the number of formulas recorded in the locality.

From the same area but extending widely to the south reaching Murcia (Table 9), the *gitam* stands out (Figure 6). It is made with *Dictamnus hispanicus* and the distribution of this liquor coincides practically with that of the species in the East of Spain. In the upper part of the production territory, these two liquors are made with high-alcohol content spirits.

In South of Valencia close to the province of Alicante, *salvieta* liquor becomes noticeable, both in its simple formula with *Salvia blancoana* subsp. *mariolensis* and in the complex formulas where other sage species are mixed such as *S. microphylla* var. *microphylla, S. officinalis* subsp. *lavandulifolia*, *S. officinalis* subsp. *officinalis*, *S.* x *auriculata*, and *S.* x *hegelmaieri* (Martínez-Francés et al., 2017).

In the South of Alicante province, *Dictamnus* disappears and *Thymus moroderi* becomes the main species. This single liquor is called *cantueso* and it is made and also commercialized in a very small area between Alicante and Murcia.

In the area described so far, from Tarragona to Murcia, excluding Aragon, complex herbal liquors are made that we have typified as *herbero*. We collected 186 formulas, of which 88% are from the different mountainous areas of the Valencian region. In this herbero type, complex formulas of Balearic Islands are included (Table 9). The Balearic formulas lack *Dictamnus* as an essential plant, because it does not exist on the islands, but it is replaced by rue. But there is a much greater difference between the composition of these liquors between the main island, Mallorca, and the rest. In Mallorca, whose main function was to combat recurrent diarrhea, *herbero* is made mainly with Rosaceae species, and with *Tanacetum balsamita*, a species considered a panacea, which also stands out. In Menorca, Ibiza, and Formentera, their liquors include both cultivated and wild plants and the *herbero* type is more similar to the peninsular ones. The Balearic Islands local variants of *herbero* are characterized by the use of *Santolina magonica* (local endemic species), *Aloysia citriodora*, and *Rosmarinus officinalis* among others (Table 9).

*Dictamnus hispanicus* and *Santolina villosa* are present in over 60% of the 186 *herbero* formulations analyzed, mainly from Valencia and Balearic Islands (Figure 6). Other species often used in herbero are *Salvia blancoana* subsp. *mariolensis* Figuerola and *Thymus vulgaris* L.

Vermouth and absinthe, though underrepresented in our samples, are both characterized by the use of *Artemisia absinthium.* Absinthe and vermouth are linked and although there have been crops and distilleries in the interior of Castellón province, their consumption was mainly restricted to the coastal areas where it was also produced at a popular and commercial level. Both sum 28 records, belonging c 90%, to the Valencia region.

The infrequent Benedictine-like liquor, which is no longer produced, is characterized by the use of *Angelica archangelica* and a mixture of spices. Walnut wine is made, especially in Valencia but also in Aragon, Castilla-La Mancha, and Murcia regions, with *Juglans regia* tender fruits that enter also in the ratafia formulations of Catalonia. Fruit wines are often aromatized with cinnamon bark.

Cucumber spirit is a fruit liquor made in the past throughout the territory, being used as a powerful digestive, but many of the bottles found were very old. Honey spirit is also elaborated in different regions of Spain. We reported formulas of *resoli* mainly from the northeast of Castilla-La Mancha, and also a mushroom spirit is elaborated there and in Castellón (Valencia region). All of them are used as digestives.

Data collected before 2007 yielded an average number of herbal ingredients of c. 10. This allowed us to establish a preliminary geographical differentiation of formulas according to the dominance of different specific groups of plants. An obvious generational rupture, away from rural and traditional customs and a subsequent interest of return to natural lifestyles, led to homemade globalization of this type of medicinal beverage. Accordingly, formulas recorded were drastically simplified with an average number of ingredients varying between 3.2 and 5.5 depending on the 4 years period considered. Presently, they can be seen as merely “author elaborations” as digestive liquors. The average number of herbal ingredients of the formulas recorded after January 1 of 2016 is 2.9. Therefore, in almost 20 years of study, we have recorded an impoverishment of the use of plant species and, also, the medicinal uses for this type of preparation.

## Discussion

### Relevant Species

*Dictamnus hispanicus* (Figures 5, 6) is present in one-third of the formulas recorded during our study (Table 7). The species grows in the study area, nowadays, among the unmanaged scrub or in inaccessible areas of the Iberian mountains. Its distribution area fits with the elaboration area of the characteristic *gitam* and *herberos*. In Spain, leaves of *D. hispanicus* (Font-Quer, 1985; Mulet, 1991; Ríos and Martínez-Francés, 2003; Martínez-Francés and Ríos, 2005; Merle et al., 2006; Popović et al., 2014; Martínez-Francés et al., 2015) were used instead of the rhizome, contrary to what is commonly used in Central Europe (Fournier, 1947; Ivanova et al., 2004; Panesar et al., 2009; Tiţǎ et al., 2009). The parts most used in the spirits studied in eastern Spain are leaves (173), while the aerial parts including stems and flowers (if any) appear in nine and rhizomes only in two. The almost exclusive use of leaves is clearly related to the sustainability of the resource from the point of view of our informants. *D. hispanicus* aerial parts were used in teas as digestive and emmenagogue (Martínez-Francés et al., 2015; Martínez-Francés et al., 2018a).

This species has multiple medicinal uses that are treated in detail in Martínez-Francés et al. (2015). The use of *D. hispanicus* for dyspepsia and other diseases of the digestive system involves the maceration in alcoholic beverages, steeping fresh leaves, or flowering tops into spirits of 25–40% of ethanol, in simple *gitam* formulas but often in other more complex named *beatamaria* and *herberet* or *herbero*, which in recent decades have gained popularity as digestive and tonics and are used within a broad social context (Martínez-Francés et al., 2015).

The use of *Dictamnus* in herbal liquors recalls that of Alpine wormwood (*Artemisia genipi* and other spp.) and gentian (*Gentiana lutea* and other spp.) in medicinal homemade digestive liquors in the Alps reputed as panaceas but used especially against coughs and as digestive and depurative (Pieroni and Giusti, 2009). Alpine *Artemisia* species and *G. lutea* have a crucial role in the Alpine folk medicine (Mattalia et al., 2012).

*Salvia blancoana* subsp. *mariolensis* (Figure 5) is an endemic plant taxon with an area restricted to a small region in the North of Alicante province and the South of Valencia province which is present in the 20% of the formulas registered (Table 7). It has the same traditional medicinal uses as *S. officinalis* subsp. *lavandulifolia* whose distribution is much wider (Anthos, 2021), this last species being the accepted source for the Spanish sage oil (Martínez-Francés et al., 2012; Martínez-Francés et al., 2017). *S. blancoana* subsp. *mariolensis* is used to prepare a digestive liquor called “*salvieta*,” alone or mixed with other wild and cultivated sage species. Its consumption in local festivities is consolidating its social use among the youngest, becoming a potential commercial product. This species is used in herbal teas as hypotensive, detoxifying, antitussive, anticold, against gum inflammation, digestive, emmenagogue, sedative and antipyretic, and the hydroalcoholic extract as tonic and digestive (Martinez-Francés et al., 2017). There are no data on the cultivation of this plant outside the botanical gardens (Reales et al., 2004; Martinez–Francés et al., 2017).

*Clinopodium serpyllifolium* subsp. *fruticosum*, with a discontinuous area, grows on the eastern coast of the Mediterranean, present in the East of Spain and in the Italian and Balkan Peninsulas and Israel (Bolòs and Vigo, 1996). In the limestone and stony rocks where it appears, it is frequently collected both to consume in teas and to make digestive liquors. In every place it grows, it is an appreciated plant, taken for relieving stomach aches and also for the treatment of nervous system disorders (Mulet, 1991; Azab, 2016).

*Thymus moroderi* (Figure 5) is an endemic plant of southeastern Spain that it is also cultivated and used to make an industrial liquor marketed since 1867 named *cantueso* (Díaz-García et al., 2014; Marco-Medina and Casas, 2015). *T. moroderi* is a potential source of anthocyanins as food additives, with both high polyphenols content and high antioxidant activity (Díaz-García et al., 2014). Due to its wide popularity and its scarcity, it is susceptible to being adulterated with other species, such as *T. longiflorus*, *T. antoninae*, and *T. granatensis* subsp. *micranthus* between others (Díaz-García et al., 2014); however, in the present study, such type of adulteration was not recorded. Long journeys are frequent in the western and southern Alicante area during *T. moroderi* flowering season, in which the population collects the plant for consumption. In Elche *Thymus moroderi*, it is also linked to one of the most deeply rooted traditions (although in evident decline, as the festival itself is suppressed), such as that of “*fer herbetes*” in the mountains that surround the Pantano de Elche on Ascension Day. In addition to being marketed for infusion, it is also the base of the most typical liqueur from Elche (Marco-Medina, 2010; Maciá, 2016).

It should be noted that in Cuenca province a liquor also known as *cantueso* is made using *Lavandula pedunculata* instead of *Thymus moroderi*; in this case, it is macerated in spirit, and later water and sugar are added (Fajardo et al., 2007).

Another highly valued endemic thyme species is *T. piperella.* Thyme species are characterized by a strong penetrating odor, but these two species are exceptional as a seasoning in meals and as an aromatizing in spirits for their pronounced balsamic and spicy flavor (Ruiz-Navajas et al., 2012).

*Polygonatum odoratum* (Figure 5) extends along temperate regions of Eurasia and N Africa (POWO, 2021). The species is scarce in the southern half of the Iberian Peninsula (Anthos, 2021). It is cultivated as an ornamental plant with numerous varieties (RHS, 2021). In southern Aragon and the south of Tarragona, a homemade liquor is made: beatamaria, which is used in menopause-associated disorders (Martínez-Francés et al., 2018b). This plant is called beatamaria only in this area, while in the rest of Spain, the Spanish translation of Salomon’s Seal is the common name. In China, the rhizomes of *P. odoratum* are usually soaked with liquor and sugar for 6 months. Before being used, the drink is strained with gauze. When drinking frequently, it is supposed to eliminate fatigue, moisten skin, and make one beautiful. The rhizomes are also used as herbal tea (Wujisguleng et al., 2012). The rhizome of *P. odoratum* is known as “*Yuzhu*.” It has the functions of nourishing Yin, clearing heat, and helping produce saliva. It can be used for the treatment of lung diseases, cough, diabetes, and indigestion. Different types of compounds have been isolated from the rhizome cholestanol saponins, furostanol saponins, spirostanol saponins, triterpenoid saponins, polysaccharides, and homoisoflavanones (Zhao et al., 2017). Pharmacological studies validate its antihyperglycemic activity, anti-inflammatory and expectorant properties in the respiratory system, immunostimulant activity, its induction to osteoclast differentiation, and the apoptosis in some human cancer (Xiao et al., 1990; Guan-Fu, 1991; Lin et al., 1994; Youn et al., 2008; Shu et al., 2009; Tai et al., 2016).

Wormwood species are native from warm regions of Europe, Asia, and North Africa (Obón et al., 2018a). In our study area, two of them, *Artemisia absinthium* and *A. arborescens*, are used in absinthe liquor and vermouth but also as an ingredient of herbero and ratafia. Absinthe has been commonly used in coastal areas as a malaria remedy, macerating wormwood in highly concentrated alcohol, in some cases near to 80% in volume (Pielech-Przybylska and Balcerek, 2019). But a complex absinthe preparation, called Vermouth, a wine-derived aperitif widely consumed (Morata et al., 2019), is also elaborated in some local distilleries of the studied territory. This aromatized liquor with a bitter taste is prepared from a base of white wine, fortified with wine spirit, colored by caramel, and aromatized with several dried herbs, spices, or their extracts (Panesar et al., 2009; Morata et al., 2019). Locally, *Centaurium quadrifolium* subsp. *barrelieri*, from Gentianaceae family, is also used in Alicante in liquors and vermouths as quina (*Cinchona officinalis*) preparations in Balearic Islands.

*Aloysia citriodora* is a widely cultivated species in our territory, although its American origin. It is an excellent organoleptic aroma and flavor corrector and also used for relieving mental stress, to aid sleep and for symptomatic treatment of digestive complaints (Vanaclocha and Cañigueral, 2019), being used in teas and liquors frequently in this area and, for instance, in Cuenca province (Fajardo et al., 2007).

*Sideritis hirsuta* (Figure 5) and *S. tragoriganum* subsp. *tragoriganum* are the most used species of this genus in herbal formulas. Highlighting the high number of genus *Sideritis* endemic species in the Southeast of Spain (Rivera and Obón, 1997), only the use of four species and one hybrid was recorded in this study. These are often used in teas for their medicinal properties (Obón et al., 2018b; 2018c).

*Eryngium campestre* roots and aerial parts are collected for use mainly in *herberos*. It is considered an essential plant, along with *D. hispanicus*. According to popular knowledge, it is added to liquors to counteract the toxicity of the Rutaceae plant. When *Dictamnus* cannot be found, it is usually replaced by another *Ruta* species and the same happens for *E. campestre*, which can be replaced by other Umbelliferae species, such as *Foeniculum vulgare* subsp. *piperitum*, *Ferula hispanica, F. tingitana,* and *Thapsia villosa*.

Although irrelevant in terms of frequency, the presence of two fungi species (*Cantharellus cibarius* Fr. and *Tuber melanosporum* Vittad.) and hen eggs (*Gallus gallus* (Linnaeus, 1758)) in the samples is worthy of mention. Mushrooms are used in liquors within the context of Traditional Chinese Medicine (Mizuno et al., 1995) and in other cultures such as in traditional medicine of Madeira (Portugal) (Rivera and Obón, 1995).

### Plant Collection and Conservation Issues

There is a relevant aspect regarding the collection of herbs. Those liquors that are made from species living far from their homes and in almost inaccessible places require our informants to travel great distances and time to get them. In *herberos* formulations, *Dictamnus hispanicus* is the most sought for, but the presence of other species (Table 7) such as *Asperula aristata*, *A. cynanchica*, *Centaurea boissieri* subsp. *mariolensis*, *Centaurium quadrifolium* subsp. *linariifolium*, *Chiliadenus glutinosus*, *Clinopodium alpinum*, *Inula montana*, *Leucanthemopsis pallida*, *Mentha aquatica*, *Saxifraga longifolia*, *Stachys heraclea*, or *S. ocymastrum* (Menéndez et al., 2018) depended on the exhaustive search in the field. Thus, the presence/absence of such a rare species inform us of the age of liquor since nowadays they are infrequent and thus no longer available.

Usually, women have been in charge of family health care, and their presence at home was continuously required, so they could not be absent from home for a long journey. That is why men are responsible for making these herbal liquors and therefore, the transmission of this knowledge is patrilineal, from father to son (Agelet and Vallès, 2001) involving some type of restrictions for the transmission outside this circuit. For this reason, data collection has not been easy, because formulas are a familiar secret and also men refused to give the recipe to a woman. In other places, such as the Balearic Islands, where the collection of herbs is limited to family gardens and nearby areas, these medicinal liquors are made by women, and the transmission is from mother to daughter.

In different parts of the studied area, we find the same problems of sustainability for some herbal resources, especially those from the most endemic and sparse species such as *Dictamnus*, *Polygonatum*, *Saxifraga*, and *Sideritis* species, similarly occurring with Alpine wormwood (*Artemisia genipi, A. glacialis,* and *A. umbelliformis*) (Pieroni and Giusti, 2009; Cornara et al., 2014).

*Dictamnus hispanicu*s is distributed on the eastern side of the Iberian Peninsula, but it apparently exhibits a very irregular abundance, being a globally rare autochthonous species. According to Anthos (2021), its western Iberian limit reaches the provinces of Granada, Jaén, Ciudad Real, Cuenca, and Guadalajara, progressively approaching the sea towards the N, where it reaches those of Huesca, Lleida, and Girona (Belda et al., 2017). However, the presence of the species in one territory does not suppose the use. In the exhaustive ethnobotanical research in the upper Guadiana River area (Ciudad Real and Albacete provinces), *D. hispanicus* uses were not recorded, neither as a medicine nor in herbal liquors despite being a relatively abundant species (Rivera et al., 2019). The elaboration of the herbero has led to an overcollection of certain species that have seen their biology modified by a change in the use of the territory. This is the case of *timó real*, *Dictamnus hispanicus,* a species with which the Torretes Research Station is carrying out a recovery project (Ríos and Martínez-Francés, 2008).

*Thymus moroderi* appears in the 2008 Red List of the Spanish vascular flora as “near threatened species” (NT) (Moreno 2008; Moreno, 2011). The main threat to this species is its collection for the manufacture of liquor and the preparation of herbal teas, as well as other local uses. Fortunately, it is a good first colonizer, like all the thyme in the Pseudothymbra section, which invades abandoned crops. Flora Protegida (2021) suggests that collection should be strictly regulated and the diversification of this activity with other species (*T. funkii* and *T. membranaceus*) (thus adulteration!), as well as establishing a minimum protected area. Since 2013, some “*in vitro*” or nursery reproduction projects have been developed, as it has been the case of Raúl Agulló from Elche. Cultivation trials are being carried out at the Camp d'Elx, promoted by the *Associació per al Desenvolupament del Camp d'Elx* (ADR) with the support of the Valencian Institute of Agrarian Research (IVIA) of the Generalitat. Three farmers initially offered to plant cantueso, but after the initiative spread, there are currently seven experimental plantations that exist in Camp d'Elx, including some 1,500 plants. The project has also been joined by the company Salas y Sirvent (SYS), which markets cantueso liquor, and which also has its own plantations (Marco-Medina, 2010; Maciá, 2016).

### The Solvents

Currently, the most common maceration bases used are spirits flavored with aniseed (*Pimpinella anisum*) or star anise (*Illicium verum*) and marketed in local warehouses. There is a multitude of traditional aniseed preparations distributed throughout the European continent (France, Italy, Turkey, Greece, etc.) and the areas under its cultural influence (Central and South America) (Pielech-Przybylska and Balcerek, 2019).

The Valencian region also participates in this tradition and there are numerous distilleries that distribute aniseed of various kinds to herbal liquor makers. These distilleries, almost all, are located within the point of maximum production of homemade and traditional *herberos* (Martínez-Francés and Ríos, 2005). Before the proliferation of distilleries, the use of homemade alcoholic distillates was very common in mountainous regions. People took advantage of winemaking residues or the entire harvest in the highest areas with low-quality wines to produce their spirits with high ethanol (between 36 and 70°).

The production technology of these spirits is widespread in the Mediterranean area. It is based on the maceration and/or distillation or redistillation of alcohol in the presence of seeds or other plant parts. Natural distilled extracts of anise seeds may also be added (Pielech-Przybylska and Balcerek, 2019). Currently, the origin of the alcohol of this aniseed spirit is molasses (*Beta vulgaris* subsp. *rapa* and *Saccharum officinarum*) and contains between 35 and 50% of ethanol in volume (Jurado, 2004) (although after soaking the herbs this content slightly decreases).

Levantine vineyards are mainly modern. Jumilla or Utiel and Requena wine regions hardly produced wines for self-sufficiency until the second half of the 19th century when they became large wine-producing areas. But there were important and deep-rooted liquor factories in the Valencian and Murcia regions (Plasencia and Villalón, 1999) despite the shortage of vineyards. The prohibition of this activity in the first half of the 20th century throughout Spain brought with it the dependence of herbero makers on legalized distilleries, which probably reduced the initial diversity of this product. Because home distillation has almost completely disappeared, many of the samples studied belong to older people or their descendants, who keep these spirits and formulas.

### The Origins of Liquors Diversity in the Area

The origin of these medicinal wines and liquors is based on the adaptation of the old herbal teas used to maintain family health to an extracting and preservative solvent such as wine and spirits. Better accessibility to medical care and medicines is the turning point for the abandonment of these practices. In many other traditional resources, their demise is imminent. This practice of making medicinal alcoholic beverages has been maintained over time thanks to its social use in popular festivals and festivities, although both the formulas complexity and their medicinal application have been distorted (Martínez-Francés and Ríos, 2009). However, Plotkin (2021) in his review underlines the ancient tradition of macerating herbs in wine for confectioning medicines that were externally applied or orally administered by ancient Mesopotamians, Egyptians, or Phoenicians; thus, part of these formulations may be independent of herbal teas. Thus, likely herbal teas do not always preced medicinal wines and liquors, and often it was the contrary (Obón et al., 2021). Data compiled by Plotkin (2021) suggest the antiquity of the discovery that hydroalcoholic extraction improves the extraction of active compounds and thus the effectiveness of the medicine and it should have been the main reason for macerating herbs in wines and beers, no matter these were topically or orally administered.

The daily intake of alcohol among the population increased associated with greater availability of distillates. At the end of the 19th century and the beginning of the 20th century, wine was a cheap product and its consumption was within the reach of any economy (Beneito-Lloris, 2003). The workers drank large amounts of wine during meals and drank also herbal liqueurs before and after work. Many mothers had some homemade healing and restorative remedies that were often given to children when they were ill, which were made with an alcoholic base. The population drank spirits or other alcoholic beverages while fasting, practice to which preventive and purifying powers were attributed (Beneito-Lloris, 1993, 2003).

In Alpine regions, we find similarities in this resource and its evolution. There, these homemade liqueurs reflect an ancient use of these products that shifted from medicine towards liquors when improved economic conditions enabled rural communities to have at their disposal larger amounts of industrial alcohol (Pieroni and Giusti, 2009).

### Liquor Types and Relationships With Industry and Other Traditions

Wine preparations are the first step between tisanes and herbal hydroalcoholic macerates. Presently, recipes with wine are rare in folk medicine, mainly in areas where homemade liquor production predominates. But some essential species remain in medicinal wine preparations, both in simple formulas with *Aloysia citriodora*, *Artemisia absinthium*, *A. arborescens* (De Natale and Pollio, 2007; Fajardo et al., 2007; Obón et al., 2018a), *Cinchona officinalis*, *Centaurium quadrifolium* subsp. *linariifolium*, *Cydonia oblonga*, *Foeniculum vulgare, Juglans regia*, *Prunus* sp. pl., *Rubus ulmifolius*, *Sambucus nigra*, and *Sorbus domestica* or complex formulas (Pollio et al., 2008), many of them, flavored with spices. In the present study, we have compiled 64 recipes involving maceration in wine. More than 50% correspond to well-known walnut wine (*J. regia*), distributed practically throughout the territory studied.

The production of homemade spirits has practically disappeared, but the older informants keep their copper alembics, the witness bottles, and the knowledge about their elaboration (Figure 2). They have been made throughout the territory. The accessibility to buy these spirits in nearby warehouses and the state persecution given its illegality have favored this abandonment. We recorded 33 samples of these homemade spirits of high graduation. These have been made mainly with the remains of the pressing of grapes (*Vitis vinifera*) or with the whole harvest due to its low production and quality in mountainous areas. Special mention for spirits made with surpluses from other crops such as *Cucurbita moschata* and *Ficus carica*. Due to the harsh taste of these spirits, in many cases, they have been rectified with anise-like flavorings as *Dictamnus hispanicus*, *Foeniculum vulgare*, *Pimpinella anisum*, and *Ruta* sp. pl. and with citral-like flavorings as *Aloysia citriodora*, *Citrus* x *limon*, *C.* x *sinensis*, and *Melissa officinalis*. Other simple liquors, generally with less alcoholic and aniseed spirits, are made with species of genres such as *Clinopodium, Mentha, Myrtus, Rosa, Rosmarinus, Salvia, Thymus*, and *Syringa*.

In Mallorca (Balearic Islands), a myrtle liquor (*Myrtus communis*) is done (Tardío et al., 2018), sometimes with flowers and leaves and others with fruits and taken as antidiarrheal and digestive. Berries of *M. communis* are used to produce a sweet myrtle liqueur in Sardinia through their infusion in spirit during 2 weeks (with 40% of ethanol in volume) (Correddu et al., 2019).

Ratafia is a liquor that follows the preparations mentioned in Tarragona and that extends to the north of Catalonia and Andorra (Table 11). It seems to be the union of two herbal traditions: on the one hand, the walnut fruit liquor accompanied by spices and other species included in the treatise of Charlemagne (closer to monastic medicine) and on the other, the herbs used in popular medicine, some of them endemic. Recipes vary from simple (one to five plants) to very complex (up to 80 plants) and are claimed to be digestive and useful in gynecological disorders (Agelet and Vallès, 2001; Bonet and Vallès, 2002; Parada et al., 2011).

Other traditional aniseed spirit base elaborations, mainly with fruits, share space with herbal wines and liquors, although in this area, they are relegated to the background. More than one hundred formulas of fruit spirit type have been collected, of which 65% belong to Valencia region, 10% to Catalonia, and 8% to Castilla-La Mancha and Aragon, respectively. The main fruits used belong to the following species: *Arbutus unedo*, *Coffea arabica*, *Cucumis sativus*, *Cucumis melo* subsp. *flexuosus*, *Juniperus communis*, *Malus domestica*, *Prunus avium*, *P. cerasus*, *P. domestica*, *P. dulcis*, *P. persica*, *P. spinosa*, and *Rubus ulmifolius*. One of the most popular fruit spirits is that obtained with *P. spinosa,* and also its Navarrese name “patxaran” is well-known everywhere (Cavero et al., 2011). Liquors made with fruits actually are increasing between nouvelle makers in most of the regions where it grows.

In other areas of Spain, such as Cuenca, the inhabitants of the sierra collected various types of wild fruits to sell them to liquors industries, especially juniper (*J. communis*) and blackthorn (*Crataegus*). In parallel, the preparation of *Pacharan* with the fruits of *Prunus spinosa* is common. In this area, homemade liqueurs are prepared with a spirit base in which various herbs, spices, and/or fruits are macerated (Fajardo et al., 2007). The most characteristic liquor of Cuenca is the *resolí* that has different forms of preparation and formulas, often secret; one of them is with orange peel, aniseed, cinnamon bark, clove, river tea (*Mentha aquatica*), pennyroyal (*M. pulegium*), coffee grains, sugar, and grape pomace (Fajardo et al., 2007). The use of these liquors is as a digestive. Liqueurs made with various fruits, plums, cherries, blackberries, or walnuts, are common in Albacete. They are usually prepared with wine spirit and sugar and it is common to let them marinate for a minimum of 6 months (Rivera et al., 2006).

The number of ingredients is extremely variable, under the type of liquor known as *ratafia*, and similarly, in the *herberos* type, we can find simple formulas with three ingredients and others with increasing complexity, with over 40 ingredients. Bonet and Vallès (2002) recorded 80 ingredients at Montseny in Barcelona province within a *ratafia* type. In our study of herbal liquors, the most frequent is the single ingredient one, followed at a distance by that of two (Figure 3). Then there is a decrease in frequency as the number of ingredients increases, but presenting saw teeth that coincide with the odd numbers.

The odd numbers’ superstition presents different forms; in Europe, a tradition that goes back to the Roman authors Virgil and Pliny and to the Pythagorean doctrine says that odd numbers bring good fortune (Schimmel, 1993). For ancient Romans, odd numbers are finite, complete and absolute, and opposed to even numbers that are imperfect and unlimited (Schimmel, 1993). From the data collected during the fieldwork, the persistence of this traditional belief that associates luck to odd numbers is evident. This concept, which is widespread in the folk medical practices of the Mediterranean area, is transferred in this case to the number of plants or the number of countable parts of the plants in the herbal formulas.

### The Medicinal Role of Liquors

Diseases associated with high morbidity and mortality rates marked the public health history of eastern Spain. Between the 17th and 19th centuries, epidemics (tertian fevers, typhus, cholera, plague, and yellow fever) caused high mortality in eastern Spain. During the 20th century, influenza and tuberculosis, typhoid fever, and malaria were common. Infectious, parasitic, and digestive diseases were the main cause of mortality followed by respiratory diseases, all this associated with hygienic, sanitary, and, on many occasions, poor nutritional conditions (Beneito-Lloris, 1993; Fresquet et al., 1994; Bernabeu-Mestre, 2004).

There is fragmentary evidence that suggests a direct relationship between specific locally prevalent pathologies and the elaboration of determined herbal mixtures to be consumed in form of teas, decoctions, or hydroalcoholic extracts obtained by maceration. This connection is attributed to the different active compounds provided by the herbal ingredients (Atoui et al., 2005; Carmona et al., 2005; Egea et al., 2015; Egea et al., 2016; Obón et al., 2014; Obón et al., 2021).

Traditional *herbero* complex formulations try to respond to these pathologies, in such a form that they can be geographically grouped by territories (Table 9) with a relationship with the locally prevalent diseases (Martínez-Francés and Ríos, 2009).

*Cinchona* and *Artemisia* preparations (absinthe and vermouth) have been widely recorded until today in lowland areas with wetlands where malaria was prevalent until 1964, as being commonly consumed, while other herbal liquor and wine preparations are more common in mountain areas where malaria was less frequent. Absinthe and vermouth have been consumed in areas near the mouths of large rivers, inland deltas, small lagoons, and marshes, mainly located in Tarragona province (Catalonia) and Castellón and Valencia provinces (Valencia region) (Fresquet et al., 1994; Martínez-Francés and Ríos, 2005).

Complex herbal and cinchona preparations in Balearic Islands have been consumed to combat recurrent diarrhea and dysentery, due to the biological contamination of their drinking waters, and also malaria. It is important to note that these islands have not permanent watercourses and the water for consumption and irrigation comes from groundwater because the predominance of limestone favors infiltration (Carrió et al., 2012a; 2012b).

In the Ports Mountains (Tarragona) and overlapping with southeastern Aragon (Teruel province), two particular liquors, *gitam* and *beatamaria*, have been recorded as used in the treatment of women disorders. The first is elaborated with *Dictamnus hispanicus* and the second is with *Polygonatum odoratum*. These two plants, of scattered and sparse distribution, but with numerous active substances (Lin et al., 1994; Martínez-Francés et al., 2015; Martínez-Francés et al., 2018a; Martínez-Francés et al., 2018b), are praised by the locals. In the case of *P. odoratum*, bottles are transmitted in families to the next generation as a heritage that is linked to their identity and popular wisdom (Martínez-Francés et al., 2018b).

## Conclusion

Most of the 569 formulas recorded are recognized in the communities to be medicinal. Almost 87% of the recorded formulations are reputed as digestive and are consumed as such. However, most herbal liquors are mainly consumed in social events. Other medicinal uses recorded are as a tonic or to improve their mood, appetite stimulant, antidiarrheal (especially Balearic *herberos*), or emmenagogue (notably *beatamaria*).

We compiled an inventory of 215 taxa from 56 families, where 212 are plants, two are fungi, and two are animals. Most are local wild species (60%), followed by cultivated and only a few are imported (6%). The proportion of imported species differs along the types and styles recorded.

Aerial parts are the most frequently used plant parts followed by leaves, flowers, and fruits. Lamiaceae is by far the most widely represented plant family in terms of species but slightly surpassed by Rutaceae in the percentage of samples. This is due to the regional relevance of *Dictamnus hispanicus, Ruta* sp. pl., and *Citrus* species.

Among the 569 formulas and/or samples of wines, spirits, and liquors studied, 423 are uniquely differing in their combination of ingredients. However, these could be classified, according to their solvent and herbal ingredient composition into twenty main types among which for their cultural relevance can be highlighted: *beatamaria, cantueso, gitam, herbero, ratafia, resoli,* and *salvieta*. Their relative frequencies and distribution in the different regions show peculiar patterns linked to the different cultural traditions and diverse resources availability.

Complex formulations with over 40 ingredients coexist with others simplest with only one single herbal ingredient. But, in the almost 15 years of this study, we have recorded an impoverishment of a 70% in the diversity of plant ingredients of the formulas and, also, the loss of medicinal uses. The main causes of the partial loss of this knowledge are diverse: improvement of hygiene, nutrition, and the health system; change or loss of rural exchanges; production of homemade distillates illegalization; intergenerational transmission of medicinal knowledge failure; and overexploitation of some resources making their collection difficult.

We collected 186 complex herbal formulas mainly in Valencia region and Balearic Islands, typified as *herbero*, of which 88% are from the different mountainous areas of the Valencian region where Dictamnus hispanicus is prevalent. The Balearic formulas lack *Dictamnus* because it does not exist on the islands, but it is replaced by rue. The different historically prevalent diseases and the associated herbal remedies together with the differential availability of resources seem to have been the main conditioning factors for this high herbal liquor diversity.

This herbal liquor diversity is extraordinary when compared not only with those of the adjacent areas but also with those of the Apennines and Alpine region.

## Data Availability

The original contributions presented in the study are included in the article/Supplementary Material; further inquiries can be directed to the corresponding author.
